# Molecular dynamics simulation of an entire cell

**DOI:** 10.3389/fchem.2023.1106495

**Published:** 2023-01-18

**Authors:** Jan A. Stevens, Fabian Grünewald, P. A. Marco van Tilburg, Melanie König, Benjamin R. Gilbert, Troy A. Brier, Zane R. Thornburg, Zaida Luthey-Schulten, Siewert J. Marrink

**Affiliations:** ^1^ Molecular Dynamics Group, Groningen Biomolecular Sciences and Biotechnology Institute, University of Groningen, Groningen, Netherlands; ^2^ Department of Chemistry, University of Illinois at Urbana-Champaign, Urbana, Champaign, IL, United States

**Keywords:** JCVI-syn3A, minimal cell, Martini force field, integrative modeling, coarse grain, polyply

## Abstract

The ultimate microscope, directed at a cell, would reveal the dynamics of all the cell’s components with atomic resolution. In contrast to their real-world counterparts, computational microscopes are currently on the brink of meeting this challenge. In this perspective, we show how an integrative approach can be employed to model an entire cell, the minimal cell, JCVI-syn3A, at full complexity. This step opens the way to interrogate the cell’s spatio-temporal evolution with molecular dynamics simulations, an approach that can be extended to other cell types in the near future.

## Introduction

Biomolecular functions emerge from the molecular interactions taking place in cellular environments. Understanding each component’s role in driving cell function poses an immense challenge. For a long time, experimental techniques have been our main window into the cellular environment. By resolving biomolecular structures and probing the dynamics of biomolecular processes, both *in vivo* and *in vitro*, researchers have developed a global understanding of how a cell functions.

A limitation of these experimental techniques is the spatio-temporal resolution that they can probe, particularly within the complex and crowded environment of the cell. Molecular dynamics (MD) simulations provide a suitable alternative approach, covering the relevant length and timescales at molecular resolution, albeit over short periods of a cell cycle. Over the past decades, MD has matured into a powerful tool that functions as a computational microscope (Lee et al., 2009; Dror et al., 2012). With the advances in available computer power, including the transition from using central processing units (CPUs) to graphical processing units (GPUs), the complexity and the spatio-temporal scales of MD simulations have increased remarkably. State-of-the-art simulations, containing hundreds of millions of atoms, include dynamic models of a photosynthetic chromatophore vesicle (Singharoy et al., 2019), the nuclear core complex (Mosalaganti et al., 2022), the membranes of a mitochondrion (Pezeshkian et al., 2020), the bacterial cytoplasm (Yu et al., 2016), and a virus particle embedded in a nanoscale aerosol droplet (Dommer et al., 2022). The natural next step is, arguably, the scale of entire cells (Bhat and Balaji, 2020; Khalid and Rouse, 2020; Luthey-Schulten et al., 2022; Thornburg et al., 2022).

Creating a whole-cell model has long been a major goal in computational modeling. A computational cell will help us to resolve a more integral picture of how biomolecular interactions drive cell function since biomolecular processes operate on a hierarchy of interconnected scales. Thus, resolving the full cell function requires a holistic approach. The current state-of-the-art uses static representations of heterogeneous cell-scale structures such as cellPACK (Johnson et al., 2015; Maritan et al., 2022), genome-scale well-stirred reaction models for metabolism (Karr et al., 2012; Macklin et al., 2014; Karr et al., 2015; Breuer et al., 2019; Macklin et al., 2020), or mesoscale kinetic models that attempt to include both structural and chemical states of the cell such as Lattice Microbes (Roberts et al., 2013). These computational techniques, despite granting impressive insights into the complexity of cellular processes, are limited by the spatial resolution they can achieve.

Constructing whole-cell models requires the integration of a large amount of experimental data, i.e., relies on an integrative modeling approach (Bonvin, 2021; Luthey-Schulten, 2021; Gupta et al., 2022). Obtaining such data with high spatial and dynamic detail, particularly in living cells, is very challenging, but exciting progress is being made in elucidating the architecture and stoichiometry of cellular components at unprecedented resolutions (Reading et al., 2017; Ando et al., 2018; Cheng, 2018; Chorev et al., 2018; Christie et al., 2020; Lorent et al., 2020; Narasimhan et al., 2020; Wietrzynski et al., 2020; Štefl et al., 2020), setting the stage for spatially detailed simulations of whole cells. To showcase this possibility, we consider one of the simplest cells known to date: the minimal cell created by the J. Craig Venter Institute (Hutchison et al., 2016), a stripped-down version of a *Mycoplasma* bacterium. The current strain, named JCVI-syn3A (Syn3A), contains only 493 genes and is still able to replicate independently (Breuer et al., 2019). This cell is particularly amenable to detailed computational modeling because it is of relatively small size (measuring 400 nm in diameter), and its precise composition has largely been resolved (Breuer et al., 2019).

Here we present our ongoing effort to simulate Syn3A using the Martini coarse-grained (CG) force field (Marrink et al., 2022). The Martini force field employs a four-to-one mapping scheme, where up to four heavy atoms and associated hydrogens are represented by one CG bead. This reduction in the number of particles in the system, together with a smoothening of the potential energy surface, speeds up the simulations by about three orders of magnitude, enabling simulations of systems that approach the size of entire cells. In the case of Syn3A, about 550 million CG particles are required, corresponding to more than six billion atoms.

**FIGURE 1:**
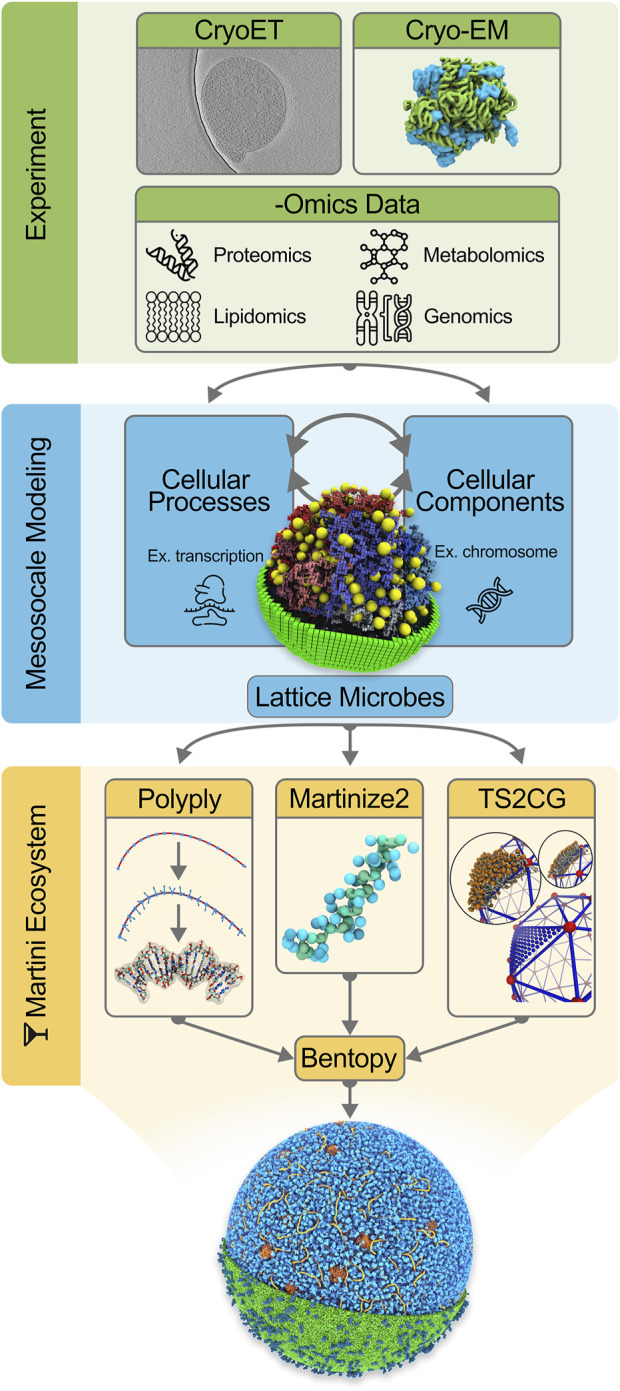
Integrative modeling workflow for building *in silico* whole-cell models. The initial step consists of collecting experimental data to inform the *in silico* modeling. Data from CryoET images [Image from EMD-23661 by Lam and Villa ref. (Gilbert et al., 2021)], Cryo-EM protein structures and -Omics experiments are incorporated into our cell models. The second stage in the workflow concerns mesoscale modeling. Here a kinetic model of the whole JCVI-syn3A [Image ref. (Thornburg et al., 2022) is used to gain quantitative insights into cellular processes and composition. As part of the final step, Martini models of the cellular components are generated using tools in the Martini ecosystem: Polyply, Martinize2, and TS2CG (Image ref. (Pezeshkian et al., 2020)]. Lastly, Bentopy facilitates the assembly of the individual molecular components in their appropriate abundances into the final molecular-resolution whole-cell model.

In the remainder of this work, we first describe the set of tools needed to construct a system as complex as an entire cell at the Martini level (the Martini “ecosystem”), including a description of the stepwise procedure to construct a starting model for Syn3A, from building the chromosome and modeling the cytoplasm to the addition of the cell envelope. We end with the prospects of actually simulating this model and discuss the potential future avenues of simulating entire cells. The integrative modeling workflow is schematically depicted in Figure 1.

## Building cells with the Martini ecosystem

Modeling cellular environments using a coarse-grained approach requires the use of a force field that incorporates enough detail to represent all biomolecules and their interactions explicitly. In this regard, the Martini force field is an excellent candidate, as demonstrated by the wide range of application studies using Martini over the past 2 decades (Marrink et al., 2022). Additionally, parameters for a large variety of biomolecules are already available, including proteins (de Jong et al., 2013), lipids (Wassenaar et al., 2015), polynucleotides (Uusitalo et al., 2015; Uusitalo et al., 2017), carbohydrates (López et al., 2009; Grünewald and Punt, 2022) and metabolites (Sousa et al., 2021; Alessandri et al., 2022). A curated collection of all parameters is available from the Martini Database (Hilpert et al., 2022).

Accompanying the Martini force field is a collection of tools that compose the software side of the Martini ecosystem (Figure 1). The primary goal of this software suite is to facilitate the construction of topologies and initial coordinates for running CG Martini MD simulations. The Martini ecosystem is currently being extended around a central framework, named *Vermouth.* Making use of a graph-based description of molecules, *Vermouth* implements a unified handling of processes frequently used in Martini, such as topology and coordinate generation or resolution transformation, as a lightweight python library (Kroon et al., 2022).

Proteins comprise the bulk of a cell’s organic material, composing approximately 40% of the total intracellular volume (Ellis and Minton, 2003). The number of unique proteins expressed by the cell, i.e., the proteome, can range from a few hundred to several thousand. Consequently, describing realistic cellular environments requires generating topology files for proteins of varying shapes and sizes as well as packing these into a highly crowded solution. *Martinize2*, which is built on top of *Vermouth*, is designed for high-throughput generation of Martini topologies and coordinates for proteins from atomistic protein structures. The workflow used in *Martinize2* additionally performs quality checks on the atomistic protein structures and alerts to potential problems making it ideal for such a high-complexity use case (Kroon et al., 2022). To generate dense protein solutions on a cellular scale as required for whole-cell modeling, a new software tool, called *Bentopy*, is currently under development. It uses an efficient collision detection scheme (Howard et al., 2016) to generate random packings of proteins and protein complexes within volumetric constraints. Furthermore, functional annotations of proteins can be integrated into the algorithm, biasing their spatial distribution in the cytosol based on their known biochemical function.

Constructing coordinates and input files for chromosomal DNA presents another challenge for modeling of a whole cell. Even for a comparatively small genome as that of JCVI-syn3A, approaches that rely on reading input coordinates and forward mapping such as used in *Martinize2*, become too inefficient due to the sheer size of the molecule. Instead the *Polyply* software, which was originally developed to efficiently setup general polymeric systems, can be used (Grünewald et al., 2022). Within *Polyply*, a multiresolution graph-based approach is used to efficiently generate polymer topologies, in particular for DNA, only from the sequence. In addition to topologies, system coordinates can be generated using a specialized biased random walk protocol. This tool of the Martini ecosystem has successfully been applied to model dense polymer melts and simple ssDNA viral chromosomes. At the moment, the package is being extended to handle double-stranded nucleic acids, and generate more complex DNA structures such as bacterial chromosomes.

Lastly, modeling lipid membranes has historically been a leading application of the Martini force field (Marrink et al., 2019). Simulating arbitrarily complex membranes of various sizes, geometries, and lateral heterogeneities is facilitated by *TS2CG* (Pezeshkian et al., 2020). This tool implements a backmapping algorithm that converts triangulated surfaces into CG membrane models. As a result of the method’s high level of control, the curvature-dependent lipid concentrations in both membrane leaflets can be precisely determined by the user. In addition, proteins can be inserted into the membrane together with their characteristic lipid shells [i.e., lipid fingerprints (Corradi et al., 2018)], setting the stage for building cell envelopes.

In the following subsections, we describe the application of the aforementioned tools to construct a proof of principle whole-cell simulation of Syn3A, illustrating how the current Martini ecosystem enables users to study multi-component systems at the mesoscale.

### Chromosome building

The minimal genome of JCVI-syn3A contains 493 genes and is encoded in a single circular chromosome of 543 kilobase pairs (kbps). Since the chromosome is contained inside the cell’s cytosol, the structural organization is heavily influenced by the crowded intracellular environment. Due to the size and near-uniform distribution of ribosomes present in the cytosol, the excluded volume interactions of these protein-RNA complexes are known to have a significant influence on the nucleoid organization (Mondal et al., 2011).

The nucleoid structure of Syn3A was previously modeled by Gilbert et al. (2021) based on the ribosome distribution and cell boundary determined by cryo-electron tomography. A Monte Carlo (MC) method grew the chromosome, modeled by a self-avoiding polygon, on a lattice inside the cell boundary. Each MC step ensured that no model constraints were violated, resulting in a circular genome without steric clashes with the ribosomes or cell membrane. The algorithm was validated by comparing the chromosome conformation capture (3C) maps of ensembles of simulated nucleoid configurations with experimental 3C maps. 3C maps show spatial correlations between chromosomal regions, which are spatially close but can be distant in the nucleotide sequence. Based on the features in the 3C maps, we infer that the chromosome is organized more like a fractal globule with little persistent supercoiling.

Whilst the previous chromosome modeling approach with a lattice polymer was tailored to be highly compatible with the whole-cell simulations using Lattice Microbes, we have subsequently developed a new method to generate circular chromosomes organized as fractal globules in a continuum polymer model with 10 bp monomers. The generated chromosome model is relaxed using Brownian dynamics and an energy function for modeling dsDNA as a twistable worm-like chain from (Brackley et al., 2014). In order to connect the chromosome model to a Martini-level representation, the model is transformed to a one-bead-per-base-pair resolution by spline interpolation. Rotation minimizing frames are then constructed along the chromosomal contour, providing a consistent reference to which the Martini DNA model can be backmapped (Wang et al., 2008). After adding an equilibrium twist along the frame’s tangent vector, Martini base pair templates matching the 543 kbp genome sequence are positioned along the chromosome following the local contour reference frame. By performing a short energy minimization the system is relaxed, resulting in a stable chromosome structure. The subsequent model consists of 543 kbps, which at a Martini resolution is equivalent to seven million beads. By implementing this backmapping procedure in *Polyply*, we are able to efficiently generate the coordinates for the chromosome in a force field agnostic manner. The overall chromosome building takes a matter of minutes, opening up the possibility of studying larger protein-DNA complexes like chromatin fibers and *Escherichia coli* chromosomes. The required topology files were generated from the sequence using the default *Polyply* methods.

### Cytosol modeling

In order to model the cytosol, it is essential to have a complete picture of the bacterial proteome, including both protein structures and proteomics counts. The genome reduction leading to Syn3A limits the number of different proteins that have to be taken into account by only retaining 452 protein-coding genes. This minimal genome has been extensively characterized, and only 91 genes remain without an annotated function. A recent study by Bianchi et al. (2022) uses computational analyses to further elucidate the function of uncharacterized genes and work toward complete functional characterization of the proteome. By gaining a better understanding of the function of encoded proteins, we will be able to inform the spatial distribution of proteins in our whole-cell model.

From the 452 different proteins expressed by Syn3A, 281 are characterized as cytosolic proteins, 63 as trans-membrane proteins, 42 as peripheral membrane proteins, and the remaining 66 still have an unknown localization. As part of the computational gene characterization workflow, Bianchi *et al.* modeled the protein structures of the entire proteome using AlphaFold2 (Jumper et al., 2021). *Martinize2* successfully converted all but one of the predicted protein structures (451) to a corresponding Martini model. Using *Bentopy*, the cytosolic protein models are packed into the intracellular volume alongside the chromosome and ribosomes. The number of copies of each protein is based on available proteomics data (Breuer et al., 2019; Thornburg et al., 2022); in total, around 60,000 proteins were distributed within a spherical volume with a diameter of 400 nm. Concerning the ribosomes, we used bacterial homologs that we had already generated previously (Uusitalo et al., 2017), placing 503 ribosomes in random orientations near the positions originally determined from the cryo-electron tomography map (Gilbert et al., 2021). Single-stranded RNA fragments were not included at this stage.

The next major component of the cytosol are the small molecules that, together with enzymatic proteins, participate in the metabolic pathways. In the current model, we include only the metabolites for which Martini topologies were already available, primarily amino acids and nucleotide cofactors (Sousa et al., 2021), and which are present at high concentrations inside Syn3A. The metabolite models were automatically generated from the topology files using *Polyply.* Based on available metabolomic data (Thornburg et al., 2022), 1.7 million metabolites are distributed within the cytosol, approximately 55% of the metabolite count for the complete metabolome.

### Constructing the envelope

Modeling the cell envelope of the Syn3A is a straightforward procedure since it is solely composed of a singular cytoplasmic membrane. Furthermore, experimental measurements indicate the absence of a cell capsule, drastically reducing the complexity of the cell boundary. The lipid membrane is constructed using *TS2CG* with a uniform lipid mixture across both membrane leaflets. It should be noted that since the minimal cell acquires membrane components through lipid synthesis from fatty acids and direct incorporation of lipids from its environment, the lipid composition of the cellular membrane heavily depends on the growth medium. We base our model on the lipidomics data presented by (Thornburg et al., 2022), indicating the presence of five main lipid types: cholesterol (59%), sphingomyelin (18%), cardiolipin (17%), phosphatidylcholines (4%), and phosphatidylglycerol (2%). In the absence of more detailed lipidomics data, all lipids are modeled with fully saturated palmitoyl tails. The total lipid count amounted to 1.3 million lipids.

Additionally, we randomly inserted membrane proteins into the cell membrane using *TS2CG*. From the available proteomics data, the number and types of membrane proteins are determined. While AlphaFold2 structure predictions can be used directly to model monomeric membrane proteins, experimental crystal structures are still required for the protein transport complexes. *Martinize2* is again used to generate the Martini models for the membrane proteins. For simplicity, we selected five abundant protein complexes and distributed these uniformly over the membrane. In total, 2,200 protein complexes were embedded in the cell envelope, corresponding with the expected number of membrane proteins present on the surface of Syn3A.

### Solvating and simulating the cell

**FIGURE 2:**
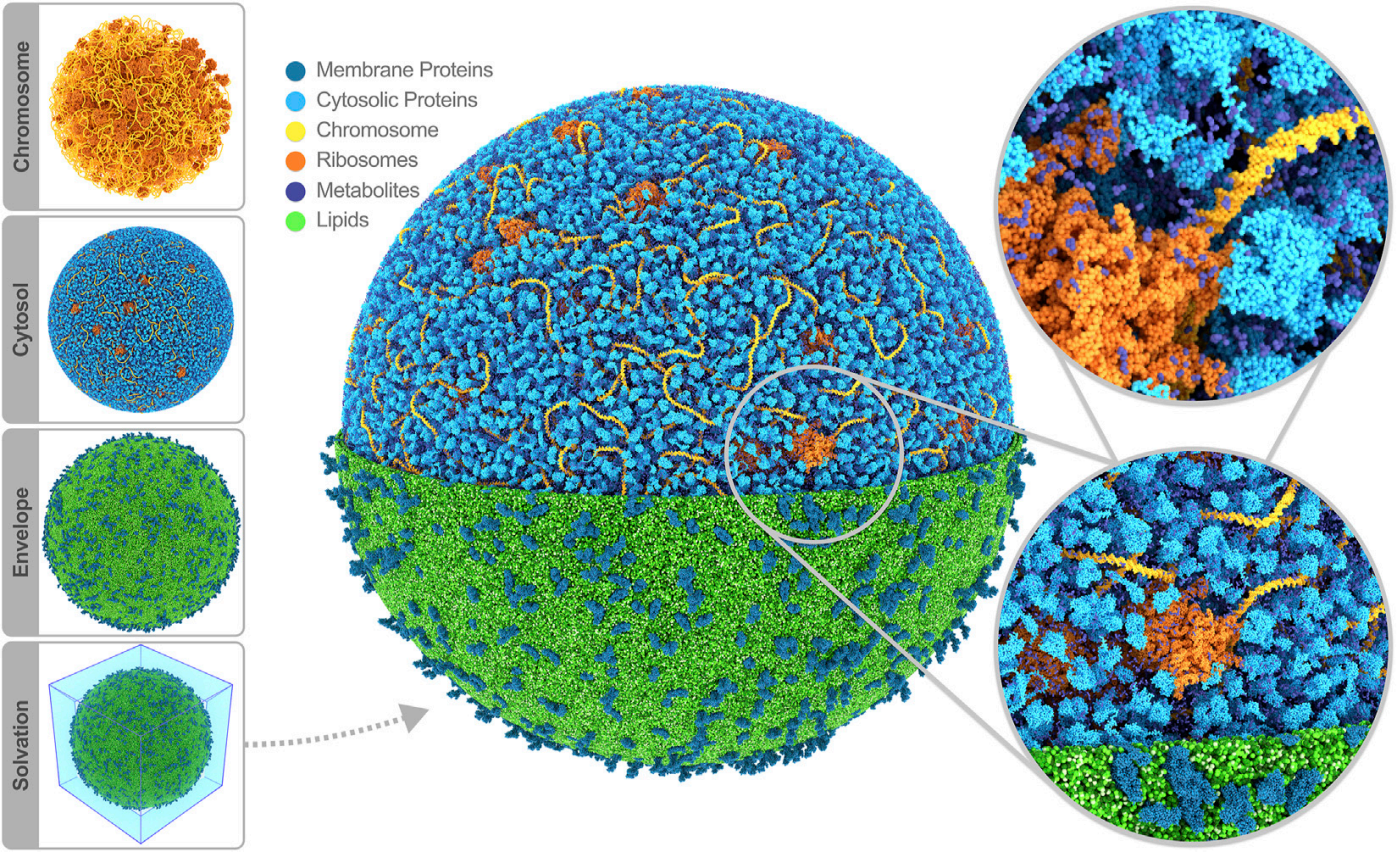
Whole-cell Martini model of JCVI-syn3A. The four stages of cell building are shown on the side. The final system contains 60,887 soluble proteins (light blue), 2,200 membrane proteins (blue), 503 ribosomes (orange), a single 500 kbp circular dsDNA (yellow), 1.3 million lipids (green), 1.7 million metabolites (dark blue), 14 million ions (not shown) and 447 million water beads (not shown) for a total of 561 million beads representing more than six billion atoms. Image rendered with Blender (Blender Online Community, 2022).

Having modeled all the cell components, the final step in constructing a starting structure for subsequent simulation is defining the periodic simulation box and solvating the system. Considering the whole-cell model’s spherical shape, a logical choice for the periodic box is a rhombic dodecahedron. To solvate, a periodic water box is tiled across the cell model, removing the water beads that overlap with the model using a collision detection scheme. The system is neutralized by placing counter ions near the highly charged components in the cytosol, i.e., the chromosome and ribosomes; the overall negative charge is substantial, amounting to 3.2 million elementary charges. As part of the solvation procedure, we also replace an appropriate number of water beads with ion beads to establish an ion concentration of 135 mM NaCl across our system, mimicking the experimental buffer. Thus, we ended up with a system containing 447 million water beads (208 million inside, 239 outside of the cell), 8.5 million sodium, and 5.3 million chloride ions. Note that Martini CG water beads represent four real water molecules. The total bead count, including all biomolecules, adds up to 561 million beads. A snapshot of the full system is shown in Figure 2.

Having constructed a starting model for Syn3A, the current challenge is to perform an actual MD simulation. At the time being, this proved to be non-trivial. Gromacs (Abraham et al., 2015), the main MD engine to run Martini-based simulations, is having difficulties handling systems comprising hundreds of millions of particles, in particular featuring large molecules such as the genome spread over multiple domains. The Gromacs developer team is aware of this problem and is dedicated to solving it. Possible other software engines to consider are ddcMD (Zhang et al., 2020) and openMM (Eastman et al., 2017), both of which are supporting Martini and offer simulation speeds comparable to those of Gromacs.

## Discussion

In the wake of a continuous rise in computing power, MD simulations have transitioned from studying idealized representations of biomolecular systems to modeling their full complexity. The culmination of this development would be simulations at the level of entire cells. As a proof of principle that we are ready to meet this challenge, we presented a model of the complete minimal cell JCVI-syn3A, constructed using the Martini ecosystem. The final simulation box comprises more than 560 million CG beads, representing over six billion atoms in the cell (Figure 2).

Before looking at the broader prospects of this endeavor, it is important to discuss a number of limitations of our approach. The current model uses the Martini 2 version of the force field since Martini models for nucleic acids, and other essential cellular components are still under development for the latest Martini 3 release. However, the methods described in this paper can be straightforwardly transferred to the latest version of Martini when validated models become available. With over 800 different bead types and a recalibrated interaction matrix, Martini 3 offers an improved framework for CG MD simulations (Souza et al., 2021). Nevertheless, inherent limitations of Martini, such as an inability to sample protein secondary conformational changes, remain. We do not anticipate that such changes are of primary importance in determining the cellular organization, but details of protein-protein and protein-lipid interactions might be affected. This problem could perhaps be resolved by using Go potentials (Poma et al., 2017; Souza et al., 2019), which are already integrated into *Martinize2* and *Bentopy.*

Even though our *in silico* cell contains more than 500 unique CG molecules and thereby presumably qualifies as the most complex system simulated to date, it simplifies the composition of various cellular components of Syn3A. Firstly, limited by the availability of Martini models for the metabolites, only a small subset is currently included in the cytosol. Future iterations of our whole-cell model will include Martini models for the complete metabolome, which comprises about 188 different compounds, and are expected to benefit from the ongoing development of dedicated automatic topology builders (Bereau and Kremer, 2015; Potter et al., 2021). Secondly, since AlphaFold2 was used to predict the protein structures of the whole proteome, only monomeric structures were initially available. Essential multimeric proteins like the ribosomes and membrane-embedded transport complexes are either left out or represented by homologous proteins with available experimental crystal structures. In the future, improved protein structure prediction algorithms will be used that also facilitate the modeling of multimeric protein structures. In addition, ongoing progress in the experimental characterization of the Syn3A proteome and lipidome, as well as the characterization of the spatial distributions of membrane proteins, will help further increase our model’s realism. A “living” list of the complete composition of our *in silico* cell can be found in our GitHub repository (marrink-lab, 2022).

Another issue is the fine-tuning of the amount of interior solvent (both water and ions), together with the lipid balance between the inner and outer leaflet. Previous works on large-scale membrane-enveloped systems (Pezeshkian et al., 2021; Vermaas et al., 2022) have shown that finding this balance is a non-trivial task. Unbalanced systems might experience strong osmotic pressures and membrane (curvature) stress, causing unwanted shape deformations all the way to membrane rupture. As a complicating factor, these effects may only appear after prolonged simulation times. Clearly, dedicated computational resources are required for the simulation of whole cells or cell organelles. The forthcoming generation of supercomputers and simulation software is becoming increasingly efficient, and billion-particle simulations have already been achieved (Jung et al., 2019; Castagna et al., 2020).

An important challenge is reaching timescales long enough to allow meaningful analysis of such large systems. Assuming dedicated computer time on current infrastructure, we anticipate that we can reach timescales of the order of 10–100 µs in the foreseeable future. Although this is typically considered a long enough simulation time for standard system sizes (e.g., a single membrane protein), it is clear that on the scale of an entire cell, we will not be able to equilibrate our system; the generated ensemble of configurations will remain dependent on our starting state. Equilibration will only happen locally, and multiple replicas will need to be generated to obtain statistically relevant data. Note that the 10–100 µs range offers a nice overlap with state-of-the-art experimental techniques. For example, advanced MINFLUX microscopy from the Hell lab enables the tracking of particles as small as 1–2 nm for 100 s of microseconds (Eilers et al., 2018; Schmidt et al., 2021). Besides, Lattice Microbes simulations of the Luthey-Schulten group (Roberts et al., 2013) use time steps of the order of microseconds, which allows for a potential feedback loop between these computational approaches.

Another major limitation is the fact that real cells operate out-of-equilibrium, driven by the import and export of nutrients and an intricate metabolic network of chemical reactions. In our approach, which is based on classical MD, we do not take this into account. We are therefore limited to studying non-reactive processes, i.e., those arising from the physical interactions among the constituents. The current composition of our cell is based on average concentrations of proteins and metabolites and thus reflects a steady-state. Coupling our classic approach with approaches taking into account reactivity, such as the aforementioned Lattice Microbes simulations or other metabolic network models (see below), in principle, could capture the non-equilibrium aspect of real cells.

Keeping these limitations in mind, simulations of the minimal cell with a molecular resolution will make it possible to study a wide range of new aspects. Modelling cellular processes and chemical transformations involves a hierarchy of interconnected scales that cannot be separated without causing artefacts. Behaviour emerging from the interaction of millions of different compounds is easily missed when systems are simplified. One might question to what extent one part of the cell affects another, given the limited timescales likely to be reached. If the various cellular subsystems act independently, one might better simulate those in isolation. To find out, one needs to simulate the complete system in addition to the smaller-scale subsystems. Our whole-cell simulation is only a first step, which will benefit from imminent improvements in high-performance computing to extend these simulations to longer timescales, up to the point where all parts of the cell may influence each other. Currently, the internal organization of the cytosol of Syn3A is primarily a black box. Our model will allow us to observe how proteins inside the cytosol interact with macromolecular structures such as ribosomes and chromosomes. Viewing the cytosol from this perspective, we can observe emerging heterogeneities and viscosity gradients, following in the footsteps of other realistic models of the cytoplasm of various cell types (McGuffee and Elcock, 2010; Yu et al., 2016; Oliveira Bortot et al., 2020). We can expect arising interaction patterns between proteins and metabolites, and probe the possible appearance of biomolecular condensates (Guilhas et al., 2020; Rhine et al., 2020).

A simulation at the level of the entire cell allows us to characterize the extent to which the cell membrane affects (and is affected by) the cellular interior. If we consider a membrane zone with a thickness of 30 nm (∼20 nm of the membrane together with its embedded proteins, plus another 10 nm layer underneath), 40% of the total cell volume is part of this membrane zone. Our simulations will provide detailed insights into the nature and extent of depletion or crowding layers, and into the level of heterogeneity inside this membrane zone, providing information on the extent to which compounds are either enriched or depleted near the cell surface (Nawrocki et al., 2019). A full-cell membrane model might explain why the minimal cell grows on a diet of both saturated and unsaturated fatty acids, but not on a diet of just saturated ones as observed in lipidomics experiments from the Saenz lab (private communication). A related question is why the cell membrane contains such a high percentage of cholesterol (20%–60% dependent on growth medium); this is uncommon for bacterial membranes although generally *Mycoplasma* do contain some cholesterol for membrane stability.

Of special interest is the potential existence of dynamic highways, i.e., regions in the cell with greater mobility of the constituents, which may arise from crowding effects or liquid-liquid phase separation phenomena, or may be induced by proximity of the cell membrane. Such dynamic highways could be important in regulating transport in an otherwise glassy state of the cytoplasm. For regions of the cell showing particularly interesting behaviour, smaller systems can be extracted with the advanced *TS2CG* tool and simulated for extended timescales to increase the statistical relevance. Besides passively studying the cellular environment, holistic cell modeling poses the ideal computational sandbox in which we can introduce new components to the cellular environment. For instance, elucidating the non-specific interactions between the cytosol and drug candidates and showing how drug-receptor interactions affect the entire cell instead of just the receptor site.

Using a multiscale modeling approach, we could potentially explore cell dynamics at various stages in its life cycle. Compared to MD simulations, other low-resolution modeling approaches can more broadly explore timescales of several orders of magnitude longer. Integrating other computational models will make it possible to sprout MD simulations in interesting regimes observed with the lower-resolution models. The primary computational method we will focus on integrating into our framework is the whole-cell fully dynamical kinetic model developed by the Luthey-Schulten lab, which accounts for the metabolic pathways governing the cellular processes (Thornburg et al., 2022). By transferring structural information from the kinetic model into our high-resolution model, it will be possible to paint a more detailed picture of the cell’s internal organization and dynamics at specific points of the cell’s life cycle, including during cell fission.

Since most of the tools in the Martini ecosystem are force field agnostic, the workflow can also be applied to generate all-atom whole-cell models. Given the substantial increase in associated computational costs, it might be a wiser approach to only sample smaller subsystems at the all-atom level. These could be straightforwardly obtained from backmapping representative regions taken from the whole-cell CG model. A number of such backmapping tools, optimized for Martini, already exist (Louison et al., 2021; Vickery and Stansfeld, 2021; López et al., 2022).

A final challenge lies in the analysis and interpretation of the complex high-dimensional massive data that will be generated. Clearly, it will be impossible to perform a comprehensive analysis on a whole-cell trajectory, and one needs to focus on specific research questions. However, the trajectories can nowadays be easily shared with the broader community *via* dedicated open-access repositories such as Zenodo (https://zenodo.org/), allowing others to perform whatever additional analysis they fancy. One can also envision the usage of data reduction schemes to efficiently analyse the whole-cell simulation. One possibility is storing only centers-of-mass movement of the non-aqueous components, which would facilitate the analysis of diffusional behavior, for instance. Another approach would be using a voxel-based method (Bruininks et al., 2021) to dynamically segment the whole-cell model into similarity regions, e.g., membrane periphery or chromosomal region. The system segmentation would allow for efficient quantitative comparison of the cytosolic properties within and between distinct regions of the cell. Furthermore, machine-learning can be invoked to extract interaction patterns and other emergent behavior that might be missed by standard analysis tools (Noé et al., 2020; Wang et al., 2020; Kaptan and Vattulainen, 2022). We foresee that our data sets will generate novel ways of dealing with this unprecedented level of complexity.

In conclusion, we presented a roadmap toward whole-cell MD simulations, illustrated with the construction of the first MD model of an entire cell using our Martini ecosystem. The model represents a next level realized with the computational microscope, providing a complete picture of the cell and making it possible to relate molecular structures and interactions to cellular function directly. In the long term, our computational framework will enable us to study a wide variety of mesoscopic systems, possibly informing the design of fully synthetic cells (Olivi et al., 2021) and modeling cells with more complex internal structures.

## Data Availability

The datasets presented in this study can be found in online repositories. The names of the repository/repositories and accession number(s) can be found below: https://github.com/marrink-lab/.
